# A new scoring system to predict the success of single-dose methotrexate therapy in ectopic pregnancy

**DOI:** 10.1590/1806-9282.20250639

**Published:** 2025-10-17

**Authors:** İbrahim Kale, Cem Yalçınkaya, Gözde Konak, Ahmet Zengin

**Affiliations:** 1 Umraniye Training and Research Hospital, Department of Obstetrics and Gynecology – Istanbul, Turkey.

**Keywords:** Ectopic pregnancy, Methotrexate, Treatment outcome, Predictive value of tests, Scoring system

## Abstract

**OBJECTIVE:**

Methotrexate remains the most commonly used agent for the medical management of ectopic pregnancy. The aim of this study was to develop a novel scoring system to predict the efficacy of single-dose methotrexate treatment in ectopic pregnancy.

**METHODS:**

In this retrospective study, information on patients diagnosed with ectopic pregnancy between January 2012 and December 2023 was obtained from the hospital information system. Notably, 156 patients received a single dose of methotrexate for ectopic pregnancy treatment and met the study criteria. Almost 117 of those who were successful in single-dose methotrexate treatment constituted the successful methotrexate treatment group. The other 39 cases who failed single-dose methotrexate treatment constituted the failed methotrexate treatment group. A new scoring system was developed based on six parameters, including serum human chorionic gonadotropin level, gestational week, and ultrasound findings.

**RESULTS:**

According to the scoring system, the total score was significantly higher in the group in which methotrexate treatment failed compared to the group with successful treatment (4 points versus 2 points, respectively, p<0.001). In receiver operating characteristic analysis, the optimal cut-off value for the scoring system was determined as 2.5, with 92% sensitivity and 78% specificity (AUC=0.804, 95%CI 0.731–0.877). The overall success rate of single-dose methotrexate treatment was 75%. However, when only patients with a score below the cut-off value of 2.5 were considered, the success rate of single-dose methotrexate treatment in ectopic pregnancy increased to 90.5%.

**CONCLUSION:**

This new scoring system significantly enhances the ability to predict outcomes of methotrexate therapy, allowing for faster decision-making regarding surgical intervention.

## INTRODUCTION

Ectopic pregnancy is the implantation of a fertilized ovum anywhere outside the uterine cavity and occurs in about 2% of all pregnancies^1^. The most common site of ectopic pregnancy is the fallopian tubes. Non-tubal ectopic pregnancies constitute less than 10% of all ectopic pregnancies and occur as cervical, ovarian, and cesarean scar pregnancies^2^.

Expectant management, medical treatment, and surgical approaches are options for the treatment of ectopic pregnancy. Hemodynamically stable ectopic pregnancy cases with an unruptured ectopic mass can be treated with methotrexate (MTX)^1^. Different treatment regimens are applied in the treatment of ectopic pregnancy with systemic MTX, including single-dose, two-dose, and multiple-dose regimens. The most appropriate treatment protocol is controversial, and each case should be evaluated on an individual basis. The overall treatment success of systemic MTX is reported to be approximately 70–95%^3^.

Studies have investigated the factors that influence the success of MTX therapy. Among these, the initial serum hCG level is the most extensively studied factor^4-6^. Maternal age, ectopic mass size, presence of yolk sac, fetal cardiac activity, and free fluid in the pelvis are other parameters investigated^6,7^. However, no universally accepted parameter exists to predict the success of single-dose MTX therapy. Therefore, this study aims to develop a scoring system to predict the likelihood of success in single-dose (50 mg/m^2^) MTX treatment for ectopic pregnancy.

## METHODS

In this retrospective study, data were obtained from the hospital information system for patients diagnosed with ectopic pregnancy at the Department of Obstetrics and Gynecology, Umraniye Training and Research Hospital, Istanbul, Turkey, between January 2012 and December 2023. During this period, 278 patients were diagnosed with ectopic pregnancy. Of these, 84 underwent surgery for rupture at diagnosis, 5 received expectant management, and 6 were treated with multiple doses of MTX. Additionally, 7 cases with unknown last menstrual period, 10 pregnancies of unknown location, and 10 cases with unknown MTX outcomes were excluded. The final analysis included 117 patients successfully treated with single-dose MTX and 39 who experienced treatment failure.

Ectopic pregnancy was diagnosed based on elevated serum hCG levels, an empty uterine cavity, and a visible ectopic mass on transvaginal ultrasound. When the uterine cavity was empty and no visible ectopic mass was present, diagnostic uterine curettage was performed. Cases showing no trophoblastic villi on frozen section were classified as ectopic pregnancies. If a frozen section was unavailable, ectopic pregnancy was diagnosed when serum hCG levels plateaued, increased, or did not decrease adequately one day after curettage. Cases receiving single-dose MTX without diagnostic curettage (pregnancy of unknown location) were excluded.

Gestational age was calculated based on the last menstrual period; cases with uncertain dates were excluded. The size of the ectopic mass was measured by averaging the two largest perpendicular diameters on transvaginal ultrasound. Masses without a visible ectopic pregnancy sac were excluded from evaluation. Hemogram parameters were obtained at the patient's initial visit, and serum hCG levels were measured both at admission and immediately before MTX administration.

A novel scoring system has been developed to predict the success of single-dose MTX treatment in ectopic pregnancy. In this scoring system, we incorporated factors known to influence the success of single-dose MTX treatment, such as gestational age, ectopic mass size, presence of a yolk sac, fetal cardiac activity, and serum β-hCG levels as key determining parameters. The parameters and scoring method used in the development of the current scoring system were adapted from the study published by Yeniocak et al. in 2024^8^. The parameters and corresponding scores used in this scoring system are as follows:

- If the gestational age at diagnosis is <5 weeks, 0 points; if ≥5 weeks, 1 point.
- If an ectopic mass is observed on USG, 1 point; if not observed, 0 points.
- If the ectopic focus mass on USG is <30 mm, 0 points; if ≥30 mm, 1 point.
- If a yolk sac is observed, 1 point; if not observed, 0 points.
- If fetal cardiac activity is detected, 1 point; if not detected, 0 points.
- Based on the β-hCG level at diagnosis, <2,000 mIU/mL: 0 point, 2,000–5,000 mIU/mL: 1 point, ≥5,000 mIU/mL: 2 points.

Serum β-hCG levels exceeding 5,000 mIU/mL and the presence of fetal cardiac activity at the ectopic site are well-established predictors of MTX treatment failure. However, patients with these characteristics were also included in the study because they initially declined surgical intervention. Each participant was scored according to these parameters at the time of diagnosis, and the total point was recorded as the final score obtained from the scoring system.

The Local Ethics Committee of Umraniye Training and Research Hospital, Istanbul, Turkey, approved this study (Ethics Committee Approval No: B.10.1.TKH.4.34.H.GP.0.01/33, Date: 09/02/2024). The study protocol was maintained by the Declaration of Helsinki.

### Statistical analysis

Statistical analyses of the study were performed using the Statistical Package for the Social Sciences (SPSS) 29 program. Kolmogorov-Smirnov test and Box plot graphics were used to evaluate the conformity of the data to normal distribution. An independent t-test was used to assess variables that showed normal distribution. The Mann-Whitney U test was used to evaluate variables that did not show a normal distribution. The chi-square test was used to compare qualitative data. The receiver operating characteristic (ROC) curve was used to determine the effectiveness of the scoring system and the significance threshold in predicting the success of single-dose MTX treatment in ectopic pregnancy. Statistical significance was accepted as p<0.05 for all values.

## RESULTS

The groups with successful MTX treatment and those that failed MTX treatment were similar in terms of age, parity, and gravidity (p>0.05 for all). Both groups were comparable in terms of the history of natural abortion, history of dilation and curettage, history of ectopic pregnancy, history of cesarean delivery, and the number of previous cesarean sections (p>0.05 for all). Both groups were similar in terms of history of infertility, history of pelvic inflammatory disease, and presence of polycystic ovary syndrome (p>0.05 for all). Both groups were similar in terms of oral contraceptive pill use, copper intrauterine device use, tubal ligation status, and coitus interruptus (p>0.05 for all). The rate of menstrual irregularities was significantly higher in the group that failed MTX treatment compared to the successful group (p=0.029) (Table 1).

**Table 1:** Comparison of the group with successful methotrexate treatment and the group that failed methotrexate treatment in terms of demographic characteristics.

				Group with successful MTX treatment (n=117)	Group that failed MTX treatment (n=39)	p-value
				Mean±SD Median (min–max) n (%)	Mean±SD Median (min–max) n (%)	
Age (years)				30.8±5	31.1±5.6	0.714^a^
Parity		Nulliparous		32 (27.35)	8 (20.51)	0.397^c^
		Multiparous		85 (72.65)	31 (79.49)	
Gravidity				2 (1–8)	3 (1–7)	0.529^b^
History of natural abortion			Yes	27 (23.08)	12 (30.77)	0.337^c^
			No	90 (76.92)	27 (69.23)	
History of D&C			Yes	5 (4.27)	4 (10.26)	0.165^c^
			No	112 (95.73)	35 (89.74)	
History of ectopic pregnancy			Yes	8 (6.84)	5 (12.82)	0.242^c^
			No	109 (93.16)	34 (87.18)	
History of cesarean section			Yes	43 (36.75)	14 (35.9)	0.924^c^
			No	74 (63.25)	25 (64.1)	
Number of previous cesarean sections				0 (0–3)	0 (0–3)	0.709^b^
History of pelvic surgery (excluding cesarean delivery)			Yes	5 (4.27)	5 (12.82)	0.059^c^
			No	112 (95.73)	34 (87.18)	
History of infertility			Yes	1 (0.85)	0 (0)	0.750^c^
			No	116 (99.15)	39 (100)	
History of PID			Yes	22 (18.8)	5 (12.82)	0.392^c^
			No	95 (81.2)	34 (87.18)	
Presence of PCOS			Yes	1 (0.85)	0 (0)	0.750^c^
			No	116 (99.15)	39 (100)	
Contraception method used						
	Oral contraceptive pills			19 (16.24)	4 (10.26)	0.361^c^
	Copper IUD			9 (7.69)	5 (12.82)	0.341^c^
	Tubal ligation			0 (0)	1 (2.56)	–
	Coitus interruptus			71 (60.68)	25 (64.1)	0.704^c^
	Not using contraception			18 (15.38)	4 (10.26)	0.426^c^
	Menstrual regularity		Yes	113 (96.58)	34 (87.18)	0.029^c^
			No	4 (3.42)	5 (12.82)	

a Independent t-test;

b Mann-Whitney U test;

c Chi-square test; D&C: dilatation and curettage; PID: pelvic inflammatory disease; PCOS: polycystic ovary syndrome; IUD: intrauterine device; MTX: methotrexate; SD: standard deviation.

Both groups were similar in terms of complaints at the time of admission (p>0.05). Gestational age was significantly greater in the group in which MTX treatment failed compared to the successful group (p=0.009). The rate of cases with an ectopic mass visible on ultrasound and a yolk sac visible within the ectopic mass was similar in both groups (p=0.203, p=0.241, respectively). In the group that failed MTX treatment, ectopic mass size was significantly larger than in the successful group, and the rate of cases with fetal heart activity in the ectopic mass was also higher (p=0.008, p=0.004, respectively) (Table 2).

**Table 2:** Comparison of the group with successful methotrexate treatment and the group that failed methotrexate treatment in terms of presenting complaints and ultrasound findings.

			Group with successful MTX treatment (n=117)	Group that failed MTX treatment (n=39)	p-value
			Mean±SD Median (min–max) n (%)	Mean±SD Median (min–max) n (%)	
Presenting complaint					
	Menstrual delay		14 (11.97)	6 (15.38)	0.580^c^
	Vaginal spotting		56 (47.86)	15 (38.46)	0.307^c^
	Pelvic pain		31 (26.5)	12 (30.77)	0.605^c^
	Pelvic pain+menstrual delay		14 (11.97)	5 (12.82)	1.000^c^
	Pelvic pain+vaginal spotting		2 (1.71)	1 (2.56)	1.000^c^
	Days of vaginal spotting		8.1±6.9	5.9±4.8	0.184^a^
	Days of abdominal pain		4.2±2.7	3.5±1.4	0.234^a^
	Gestational age (week)		5.8±1.4	6.5±1.2	0.009^a^
	Ectopic mass seen on USG	Yes	91 (77.78)	34 (87.18)	0.203^c^
		No	26 (22.22)	5 (12.82)	
	Ectopic mass size (mm)		18.15±8.16	23.09±11.24	0.008^a^
	Yolk sac seen in the ectopic mass	Yes	15 (12.82)	8 (20.51)	0.241^c^
		No	102 (87.18)	31 (79.49)	
	Fetal heart activity seen in the ectopic mass	Yes	2 (1.71)	5 (12.82)	0.004^c^
		No	115 (98.29)	34 (87.18)	

a Independent t-test;

c Chi-square test; USG: ultrasound; MTX: methotrexate; SD: standard deviation.

Both groups were similar in terms of hemogram parameters, the neutrophil–lymphocyte ratio and the platelet–lymphocyte ratio (p>0.05 for all). Serum β-hCG levels at the time of admission and at the time of MTX administration, as well as the duration of hospitalization, were significantly higher in the MTX treatment failure group compared to the successful treatment group (p<0.001, for all). The median time until serum β-hCG levels became negative was similar in both groups (p=0.568). According to the scoring system, the total score obtained was significantly higher in the group with failed MTX treatment than in the successful group (4 points and 2 points, respectively, p<0.001) (Table 3).

**Table 3:** Comparison of the group with successful methotrexate treatment and the group that failed methotrexate treatment in terms of laboratory test results and the scoring system.

	Group with successful MTX treatment (n=117)	Group that failed MTX treatment (n=39)	p-value
	Mean±SD median (min–max) n (%)	Mean±SD median (min–max) n (%)	
Hemoglobin (g/dL)	11.8±1.4	11.9±1.2	0.931^a^
Hematocrit (%)	35.9±3.4	35.6±3.4	0.592^a^
White blood cell count (10^3^/μL)	8.24 (4.01–17.1)	8.27 (4.01–18.83)	0.846^b^
Neutrophil count (10^3^/μL)	5.26 (1.84–15)	5.24 (2.29–16.9)	0.854^b^
Lymphocyte count (10^3^/μL)	2.27±0.9	2.31±0.75	0.771^a^
Platelet count (10^3^/μL)	266.32±53.45	259.21±51.65	0.469^a^
Platelet distribution width	16.3 (14.5–21.3)	16.3 (14.8–22.1)	0.054^b^
Mean platelet volume (fL)	8.8±1.42	8.71±1.43	0.735^a^
Plateletcrit (%)	0.23±0.05	0.23±0.06	0.714^a^
Neutrophil–lymphocyte ratio	2.48 (0.8–20.62)	2.43 (0.49–14.08)	0.684^b^
Platelet–lymphocyte ratio	121.21 (57.96–176.19)	116.5 (42.98–263.46)	0.329^b^
Beta-hCG level at admission (mIU/mL)	1,168 (39–41,297)	3,496 (584–16,182)	<0.001^b^
Beta-hCG level at Mtx administration (mIU/mL)	1,140 (54–40,782)	3,662 (707–18,980)	<0.001^b^
Time to surgery after MTX administration (days)		8 (2–15)	–
Duration of the hospital stay (days)	5 (1–13)	8 (2–21)	<0.001^b^
Time to negative serum β-hCG (days)	20 (5–94)	20 (10–45)	0.568^b^
Total points received according to the scoring system	2 (0–5)	4 (2–7)	<0.001^b^

a Independent t-test;

b Mann-Whitney U test; hCG: human chorionic gonadotropin; MTX: methotrexate; SD: standard deviation.

ROC analysis was performed to determine the value of the scoring system in terms of predicting the failure of single-dose MTX treatment in ectopic pregnancy. Area under the curve (AUC) analysis of the scoring system for estimating the failure of single-dose MTX was 0.804 (p=0.001, 95%CI 0.731–0.877). The optimal cut-off value for the scoring system was identified as 2.5, corresponding to a sensitivity of 92%, a specificity of 78%, a positive predictive value of 56.4%, and a negative predictive value of 80%.

The overall success rate of single-dose MTX treatment in this study was 75%. However, when the patients with a score below the cut-off value of 2.5 are taken into account, the success rate of single-dose MTX treatment in ectopic pregnancy increases to 90.5% (successful in 77 cases and failed in 8 cases).

## DISCUSSION

Single-dose MTX treatment is considered an effective and reliable treatment in selected patients diagnosed with ectopic pregnancy. Based on this, clinicians have developed some scoring systems to predict the success of single-dose MTX treatment in ectopic pregnancy. In this context, the first scoring system was reported by Elito et al. in 1999. The parameters of this scoring system consisted of serum hCG levels, ultrasound images, ectopic mass size, and color Doppler findings. The authors determined a cut-off value using this scoring system to predict the success of MTX treatment. While the overall success rate of a single-dose MTX treatment in this study, which included 40 participants, was 75%, it was noted that the success rate increased to 97% at and above the cut-off value specified according to this scoring system^9^.

In another study, Kahyaoglu et al. developed a different scoring system. The parameters of this scoring system were the serum hCG level, the aspect of the image on ultrasound, the size of the ectopic mass, and the shock index value at the time of diagnosis. This study included participants receiving single- and double-dose MTX treatment. The authors reported an overall MTX treatment success rate of 72%. ROC analysis was performed to determine the predictive power of the scoring system in identifying MTX treatment failure, and a cut-off value was established. This cut-off value was determined to be 100% sensitivity and 95% specificity in detecting failure of MTX treatment with an AUC of 0.72 (95%CI 0.51–0.92)^10^.

Başkıran et al. reported two parameters (BW and BP) that they developed using serum hCG and hemogram parameters to predict the success of MTX treatment in ectopic pregnancy. The BW parameter was calculated as hCG×WBC/1,000, and the BP parameter as hCG×1,000/PLT. All patients diagnosed with ectopic pregnancy who received the first single dose and the second single dose of systemic MTX treatment were included in this study. In total, 208 participants completed MTX treatment successfully, while 25 participants experienced treatment failure. Both BW and BP were significantly higher in the MTX treatment failure group than in the successful group. ROC analysis was performed to understand the effect of BW and BP in the diagnosis of patients who will need surgery for ectopic pregnancy. The areas under the curve for BW and BP were determined to be 0.99 (p=0.01, 95%CI 0.94–0.98) and 0.94 (p=0.01, 95%CI 0.91–0.97), respectively^11^.

In 2024, a different scoring system was reported to predict the success of single-dose MTX treatment in ectopic pregnancy. The parameters of this scoring system consisted of initial serum hCG levels, endometrial thickness, presence of peritoneal fluid observed on ultrasound, presence of an isthmic ectopic pregnancy, presence of a yolk sac, and size of the adnexal mass. The authors determined an optimal cut-off value using the scores in this scoring system. Accordingly, the 2.5 cut-off value was determined to be 91.7% sensitivity and 59.7% specificity in detecting failure of MTX treatment with an AUC of 0.81^8^.

To date, four different studies have proposed distinct scoring systems for predicting the success or failure of MTX treatment in ectopic pregnancy. In these studies, participants exhibited varying clinical and demographic characteristics, and the parameters included in the scoring systems also differed.

In this study, we developed a new scoring system using objective parameters. With this scoring system, we aimed to identify patients with a high risk of failure in single-dose MTX treatment at the time of diagnosis of ectopic pregnancy. Therefore, we believe that these patients can be directed to surgical treatment without unnecessary delay and without being exposed to the systemic effects of MTX.

This study has important limiting factors. As a single-center study, the findings may not be generalizable to other patient populations and clinical practices, which could limit their applicability. Additionally, the retrospective design introduces inherent risks of data selection bias and incomplete records. The other limitation of this study is the lack of internal validation of the predictive model, such as bootstrapping or cross-validation. This may raise concerns regarding potential overfitting. Future studies with larger cohorts are warranted to perform internal and external validation of the model. The gestational week is one of the six parameters used in this scoring system. Therefore, it is an essential limitation that this scoring system cannot be used in cases of ectopic pregnancy where the gestational age is unknown.

In conclusion, this study presents a novel scoring system for predicting the failure of single-dose MTX treatment in ectopic pregnancy, incorporating serum hCG levels, gestational age, and ultrasound findings. This system can help identify patients who require surgical intervention during diagnosis, thereby preventing unnecessary exposure to MTX. However, as this scoring system is simple and open to further refinement, its clinical utility must be validated through large-scale studies before it can be widely implemented.

## Data Availability

The datasets generated and/or analyzed during the current study are available from the corresponding author upon reasonable request.
